# Epidemiology and antibiotic susceptibility of Vibrio cholerae associated with the 2017 outbreak in Kasese district, Uganda

**DOI:** 10.1186/s12889-019-7798-6

**Published:** 2019-10-29

**Authors:** Jacob Stanley Iramiot, Innocent B. Rwego, Catherine Kansiime, Benon B. Asiimwe

**Affiliations:** 1 Department of Medical Microbiology, College of Health Sciences, Makerere University, Kampala, Uganda; 2 Department of Microbiology and Immunology Faculty of Health Sciences, Busitema University, Mbale, Uganda; 3 One Health Central and Eastern Africa (OHCEA) network, School of Public Health, Makerere University, Kampala, Uganda; 4 Department of Biosecurity, Ecosystems and Veterinary Public Health, College of Veterinary Medicine, Animal Resources and Biosecurity, Makerere University, Kampala, Uganda; 5 One Health Division, College of Veterinary Medicine, University of Minnesota, St. Paul, MN USA

**Keywords:** Cholera, Antibiotic resistance, Epidemiology, Environment

## Abstract

**Background:**

Uganda is among the 51 countries where cholera outbreaks are common with epidemics occurring predominantly along the western border with Democratic Republic of Congo (DRC), Kampala city slums, Busia district which is a border town with Western Kenya, Mbale district and the Karamoja Sub-region. This report summarizes findings from the epidemiologic investigation, which aimed at identifying the mode of transmission and antibiotic susceptibility patterns of the *Vibrio cholerae* isolated in Kasese district, Uganda.

**Methods:**

A descriptive cross-sectional study was carried out between 2017 and 2018 to describe the epidemiology of the cholera epidemic in Kasese district, Uganda. Rectal swabs were collected from 69 suspected case-persons and cultured on Thiosulphate-Citrate-Bile-Salts Sucrose (TCBS™; SEIKEN Japan) agar and incubated at 37 °C for 18–24 h. The isolates were serotyped with polyvalent 01 antiserum and monovalent serotype Inaba and Ogawa antisera (Denka Seiken, Tokyo, Japan) to determine which serotype was responsible for the outbreak. Antimicrobial susceptibility testing was performed using the Kirby-Bauer disk diffusion method on Mueller-Hinton agar.

A list of discharged patients was obtained from the isolation units of Bwera hospital and Kagando hospital and the individuals were followed to the community where they live. Questionnaires were administered to a total of 75 participants who were either the cases or relatives to the case. GPS points of the homes of the cases and pictures of potential source infection were also taken and cases were mapped.

**Results:**

A total of 222 cases were recorded in the Kasese District outbreak between the month of September 2017 and January 2018 with the case fatality rate (CFR) of 1.4%. Children below the age of 14 years contributed the biggest proportion of the cases (70%) and out of these, 33% were aged below 5 years. Culture isolated 69 *V. cholerae* 01 serotype Inaba from the total of 71 samples. *Salmonella typhi* was Isolated from the other two samples which were negative for *V. cholerae*. Antibiotic susceptibility using Kirby-Bauer disc diffusion method was done on isolates from 69 participants and showed 100% resistance to Ampicillin and over 50% were resistant to trimethoprim/Sulfamethoxazole whereas gentamicin showed 100% susceptibility. Environmental assessment revealed rampant cases of open defecation.

**Conclusion:**

Though we did not culture water to confirm contamination with *Vibrio cholerae*, we hypothesize that the cholera epidemic in Kasese 2017 was sparked off by consumption of contaminated water following the heavy floods that washed away latrines into water sources in Bwera, Isango and Nakiyumbu sub-counties. *V. cholerae* was also highly resistant to the commonly used antibiotics.

## Background

Cholera has continued to be a major threat to the wellbeing of communities globally and an important indicator for poor social progress [1, 2]. About 3–5 million people annually suffer from Cholera and 100,000–120,000 lives lost [3]. Vast epidemics of cholera have occurred in Africa consistently since 1970 with case notifications accumulating to 3,762,902 [2] and 56,329 of these cases reported in 22 African countries in the year 2013. These numbers are largely under estimated owing to the non-functional national epidemiological and laboratory surveillance systems which facilitate detection of the majority of mild disease presentations [2]. Emergencies directly affect a health care system and the impact is much felt in a resource limited setting [4, 5]. Natural calamities like earthquakes, landslides and flooding, the political situation including wars, civil unrest coupled with the social-cultural behavior like poor refuse disposable and open defecation lead to endemic outbreaks of cholera [6].

Uganda is among the 51 countries where cholera outbreaks are common with epidemics occurring predominantly in Kasese, Kampala city slums, Busia district which is a border town with Western Kenya, Mbale district and the Karamoja Sub-region [7]. Kasese district is affected by regular cholera epidemics with different Serotypes and biotypes of *V. cholerae* [8]. However, antibiotic susceptibilities have not been determined. Poor hygiene and sanitation conditions and regular flooding of the low lands usually create an ideal environment for disease to spread [9]. Kasese suffers frequent annual cholera episodes and in each outbreak, death of a case is the usual trigger for the surveillance activities. On 2nd October 2017, the Uganda Ministry of Health issued an alert of the outbreak of cholera in Kasese District. This report summarizes findings from the epidemiologic investigation, which aimed at identifying the probable cause of the outbreak and antibiotic susceptibility patterns of the *Vibrio cholerae* isolated in Kasese district to the commonly used antibiotics in our setting.

## Methods

### Study design and study area

**Fig. 1:**
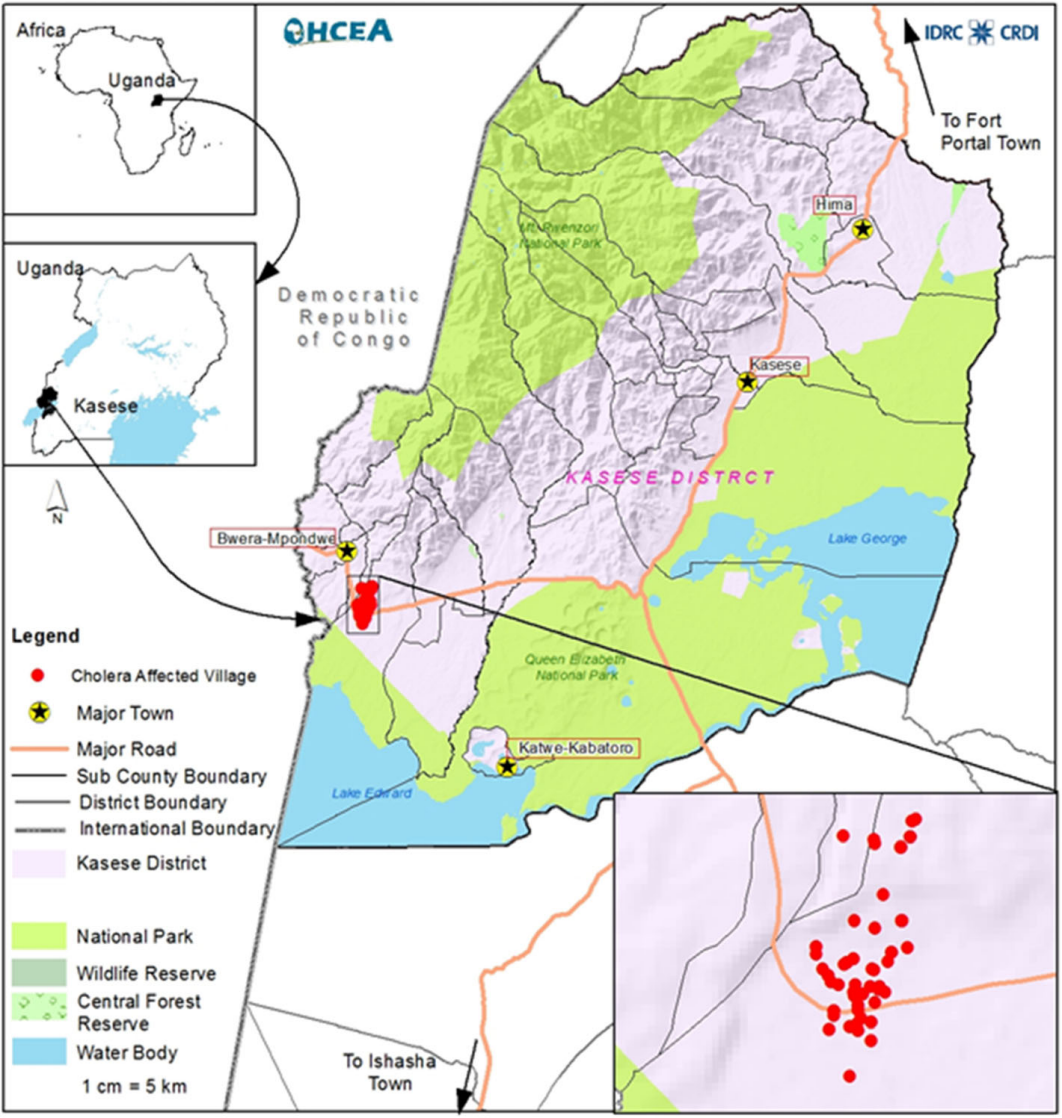
Map of Kasese district showing the study area and distribution of cases. Figure 1 is our own, drawn from the GPS coordinates taken using a hand held GPS during the study and analyzed using ARC GIS software

A descriptive cross-sectional study was carried out between 2017 and 2018 to describe the epidemiology of the cholera epidemic in Kasese district, Uganda (Fig. 1). Kasese district lies along the equator and it is bordered by the districts of Kabarole to the north, Rubirizi to the south, Kamwenge to the east and the Democratic Republic of Congo to the West. The biggest population of the district live in rural areas and practicing subsistence farming. With a population of 757,269 people and 134,037 being children below 5 years the leading cause of morbidity was malaria and acute diarrhea; which is also a major presenting symptom for cholera was the second leading cause of morbidity. Cholera ranked as the third most important cause of Morbidity [10].

### Study population and sampling

The study population comprised of both adults and children suspected to be suffering from cholera admitted in the isolation wards in Kagando hospital and Bwera hospital. According to the World Health Organization’s standard case definition: if cholera is not known to be present in the area, a case is a patient ≥5 years with severe dehydration or death from acute watery diarrhea, while during the epidemic, every patient ≥5 years with acute watery diarrhea and/or vomiting is considered a case [11]. In the community, the patients who were discharged from the isolation wards or their parents/guardians formed the study population. Consecutive sampling was carried out throughout the outbreak season until the time when the epidemic was controlled. Sample size calculation was not applicable.

### Data collection

A list of discharged patients was obtained from the isolation units of Bwera hospital and Kagando hospital and the individuals were followed to the community where they live. A total of 222 patients were recorded in the discharge book some of whom were children and these were followed to the community. We obtained telephone contacts of the care takers and worked with community health workers to locate the cases. Key questions on symptomatology and environment were asked. Major symptoms looked out for included acute diarrhea, vomiting and abdominal cramps. Environmental assessment was carried out using an environmental checklist. The key elements of the environmental checklist included; safe water, food safety, sanitation and hygiene, personal, family and school hygiene, municipal water supplies, other water supplies, solid waste disposal, disposal of excreta and treatment of waste water. We also took GPS points of the homes of the cases and pictures of potential source infection (Fig. 1).

### Laboratory detection of *Vibrio cholerae*

Self-collected rectal swabs from suspected case-persons were transported to the laboratory in alkaline peptone water medium for culture and sensitivity. ‘Case-persons’ is used in this study to mean persons with signs and symptoms matching the standard definition for the cholera case [11]. The samples were cultured on Thiosulphate-Citrate-Bile-Salts Sucrose (TCBS™; SEIKEN Japan) agar and incubated at 37 °C for 18–24 h. The isolates were serotyped with polyvalent 01 antiserum and monovalent serotype Inaba and Ogawa antisera (Denka Seiken, Tokyo, Japan). Rectal swabs were collected from 71 participants and antibiotic susceptibility done in Bwera hospital and Kagando Hospital laboratories.

### Antibiotic susceptibility testing

Antimicrobial susceptibility testing was performed using the Kirby-Bauer disk diffusion method on Mueller-Hinton agar. The *Escherichia coli* reference strain ATCC 25922 was used as a control. Isolates were tested against 7 antimicrobial drugs as follows: ampicillin (10 μg), ciprofloxacin (5 μg), chloramphenicol (30 μg), gentamycin (30 μg), nalidixic acid (30 μg), Sulfamethoxazole/trimethoprim (1.25 μg + 23.75 μg) and tetracycline (30 μg) (all Oxoid, United Kingdom). Zones of inhibition were interpreted according to the 2014 Clinical and Laboratory Standards Institute (CLSI) guidelines as resistant and susceptible [12].

### Potential sources of bias

Recall bias was the most common potential confounder as recollection of previous events might have been difficult for our respondents. However, the authors tried to minimize this by administering the questionnaires during the outbreak season to benefit from fresh memories.

### Ethical considerations

Ethical approval was obtained from the Makerere University School of Biomedical Sciences Higher Degrees Research and Ethics committee (SBS-292). Written informed consent was obtained from all study participants. Consent from parents/guardians of participants below 18 years was sought and ascent was obtained from all minors who took part in this study. Participation was voluntary.

### Data management and presentation

Data was checked for completeness to avoid cases of missing data and entered in Microsoft Excel. The variables of interest collected include; age sex, date of onset of symptoms, date of admission, duration of hospitalization, outcome and laboratory results, recovery and death. Cleaned data was exported to STATA v14 for analysis in time, place and person and presented in form of Tables and Figures.

## Results

### Epidemiological description of the 2017 outbreak

**Fig. 2:**
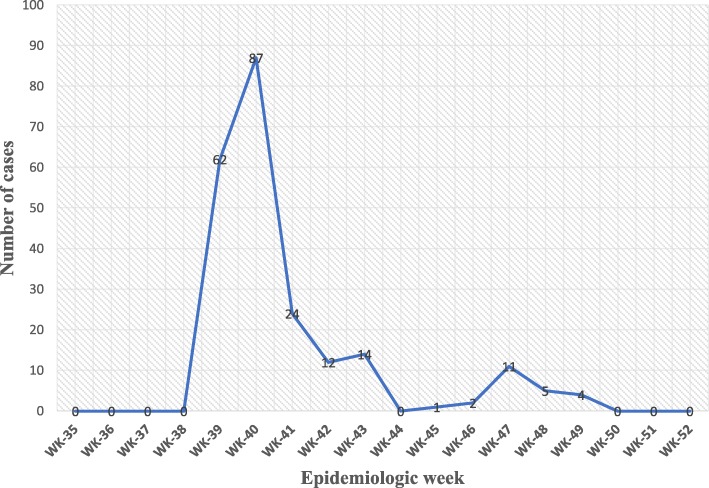
Kasese Cholera 2017 epidemic curve showing weekly notification of suspected cholera cases

A total of 222 cases were recorded in the Kasese District outbreak between the month of September 2017 and January 2018 with the case fatality rate (CFR) of 1.4%. By the time the outbreak started, the laboratory lacked necessary supplies to confirm the cases. The patients who died were two females aged 17 and 30 years and one male aged 60 years. The index case reported onset of symptoms on 23rd September was admitted on 24th September, the number of cases came to a peak at 87 in the epidemiologic week 39 and the last case was admitted in the epidemiologic week 49 (Fig. 2) before a drastic reduction in the epidemiologic week 41 due to initiation of the interventions to control the disease by the district response team. The key components of the response included; coordination, epidemiology and surveillance, laboratory identification, risk communication and social mobilization. The response strategy was adopted from the logic model for Uganda’s health sector preparedness for public health threats and emergencies [13]. Few cases continued to be reported in a lower frequency until the outbreak was fully contained by the 8th of January 2018. There were however no long term interventions put in place to prevent future outbreaks. The median age of the cases was 8 years and ranged from 7 months to 75 years. Children below the age of 14 years contributed the biggest proportion of the cases (70%) and out of these, 33% were aged below 5 years. Katojo primary school had 22 cases among the pupils and their latrine was washed away by the floods into river. The average hospital stay for the admitted cases was 4 days.

### Symptomatology and clinical picture

Questionnaires were administered to a total of 75 participants who were either the cases or a relative to the case. In instances where the case was found dead or when the case was still recovering we opted to interview a relative who was a care taker. For the cases that were clustered in one place, only one case or relative of the case was selected randomly and considered for interviews because we were likely to get similar responses since they shared common water sources, toilets etc. There was no other scientific rational for the selection criteria.

The greatest proportion of the cases (92%) were clinically diagnosed and managed without laboratory confirmation for Cholera. All the cases in this out break presented with diarrhea as the most common symptom (100), followed by vomiting (92%). Abdominal cramps were however present in only 39% of the cases (Table 1).

**Table 1:** Distribution of cholera cases by person, place, time and diagnosis in Kasese district, 2017

Characteristics	No. cases	percentage
Age group (Years)		
<5	25	33
5–14	28	37
15–24	5	7
25–34	6	8
≥35	11	15
Sex		
Male	41	55
Female	34	45
Source of Drinking water		
Untreated open water source/river	37	49
Municipal tap water	12	16
Borehole water	26	35
Duration in isolation unit (days)		
1–3	43	57
4–6	28	37
Above 6	4	5
Symptoms		
Diarrhea	75	100
Vomiting	69	92
Abdominal cramps	29	39
Type of diagnosis		
Clinical	69	92
Laboratory confirmation	6	8

### Environmental conditions in the outbreak area

**Fig. 3:**
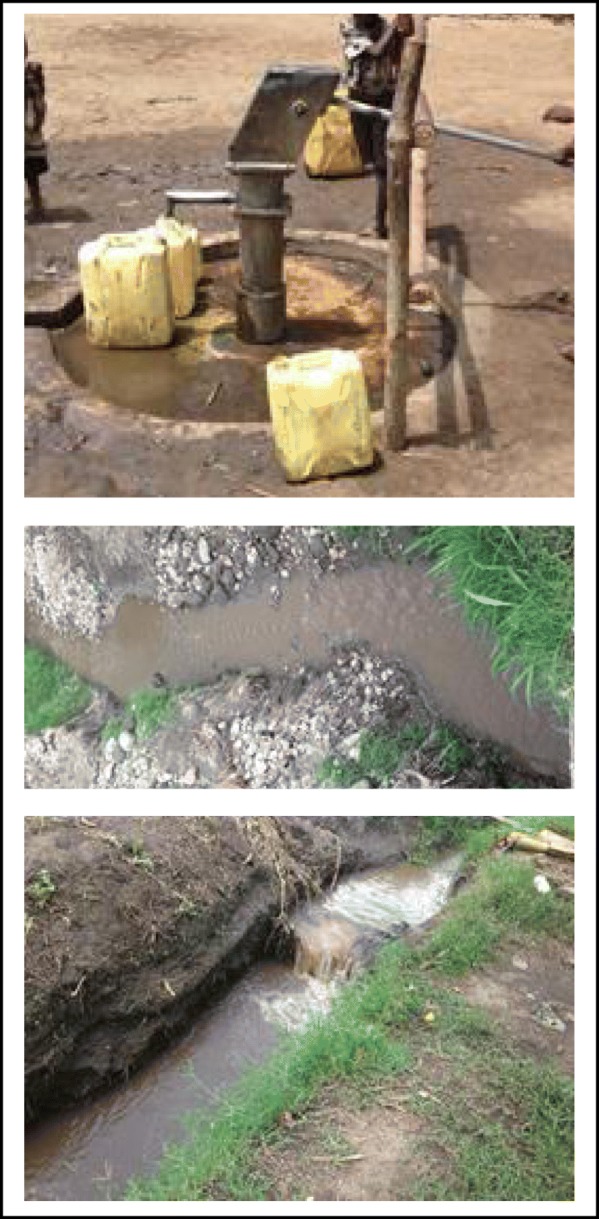
**C**ommon sources of drinking water in the affected area

Environmental assessment of the residences of the cases indicated inadequate water supply, poor sanitary conditions and unsafe disposal of solid waste (Fig. 3). All the three case fatalities were reported to consume untreated surface water with no methods employed to make the water safe for human consumption. Though we were not able to culture water samples to confirm the source of infection, we hypothesize that the cholera epidemic in Kasese 2017 was sparked off by consumption of contaminated water following the heavy floods that washed away latrines into water sources in Bwera, Isango and Nakiyumbu sub-counties.

### Water sources and safety

Drinking safe water was not a concern until the time of the outbreak with 49% (37/75) of the homesteads consuming untreated surface water. Only 16% (12/75) of the homesteads reported consuming municipal tap water while the rest, 35% (26/75) consumed borehole water. None of the participants reported boiling drinking water or use of chemi-sterilants before the outbreak period. During the mapping exercise, we did not find any piped (tap) water in the entire outbreak zone and there was only one borehole sited in the whole community. The main source of water was river Kiyanzi which is untreated surface water, visibly turbid with on-going sand mining activities.

### Other sanitary conditions in the homes of the cases

**Fig. 4:**
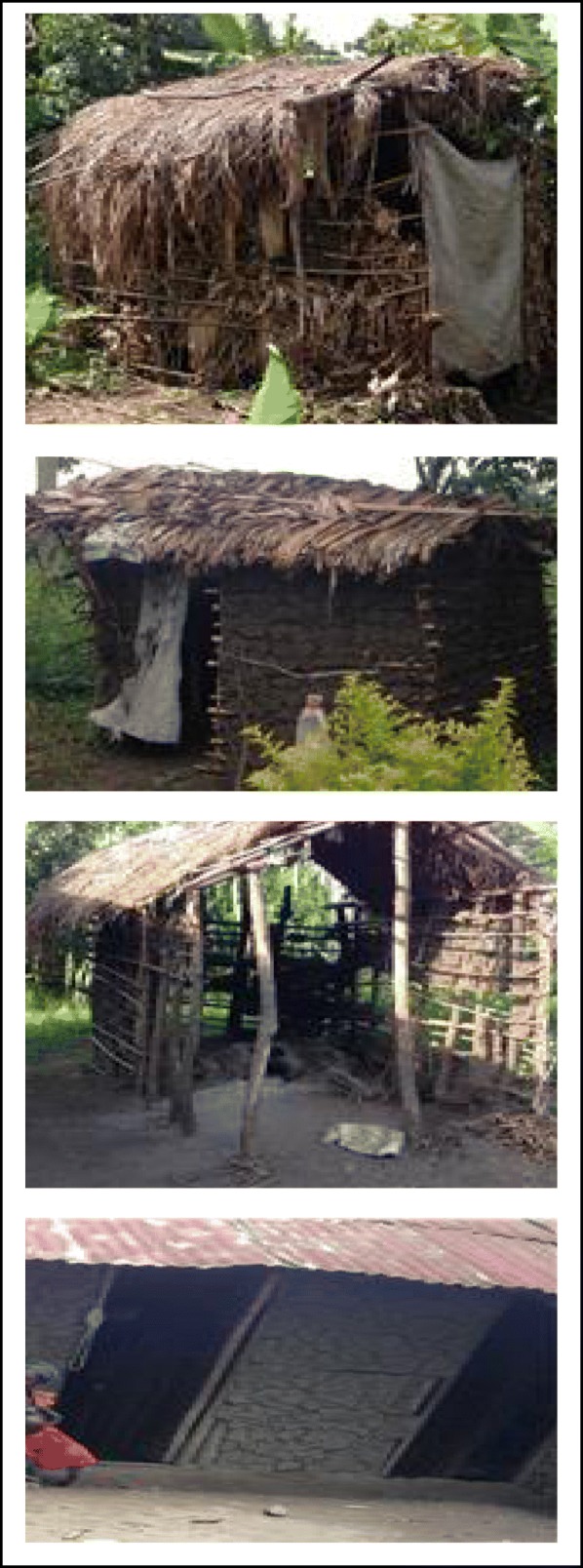
Example of sanitary conditions in the homesteads of the cases

Whereas most households in the outbreak area had poor pit latrines, there were rampant cases of open defecation. Most of the pit latrines were unimproved (Fig.4) and some were washed away by heavy floods into the river and this is what sparked of the outbreak. Hand washing facilities were not observed at the pit latrine area, a good indication for poor hand hygiene. Most of the toilets and kitchens were built as temporary structures using mad, banana fiber or grass which compromises food hygiene.

Besides the poor management of the human excreta, there was generally poor management of solid waste in the entire community. River banks were the dumping sites for garbage. The cooking areas were not safe for preparation of food and when it rained, the whole area was covered by mud. Most houses of the cases were made of mud walls and most times, especially during flooding, rain water entered the houses.

The most affected people were from Bukonzo West constituency, an area that neighbors the Democratic Republic of Congo. This *V. cholerae* outbreak was epidemiologically linked to consumption of untreated surface water after heavy flooding leading to a number of pit latrines washing away into the water according to our environmental assessment report and case and/ care taker interviews. Water from this river is consumed without treatment and therefore exposing people to cholera and other water borne diseases.

### Antimicrobial susceptibility

**Fig. 5:**
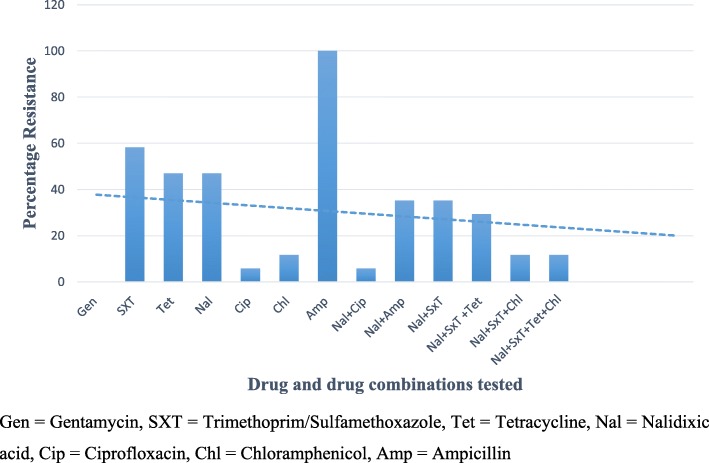
Antibiotic Resistance Patterns for *Vibrio cholerae* in Kasese District

Antibiotic susceptibility using Kirby-Bauer disc diffusion method showed 100% resistance to Ampicillin and over 50% were resistant to Trimethoprim/Sulfamethoxazole. Drugs like tetracycline which is among the recommended drugs in the clinical guidelines had close to 50% resistance whereas gentamicin showed 100% susceptibility. In addition, drugs like chloramphenicol and ciprofloxacin showed low resistance rates (11.76 and 5.9%) respectively. The general trend also showed increased susceptibility to combination therapy as opposed to mono-therapy (Fig. 5).

## Discussion

**Fig. 6:**
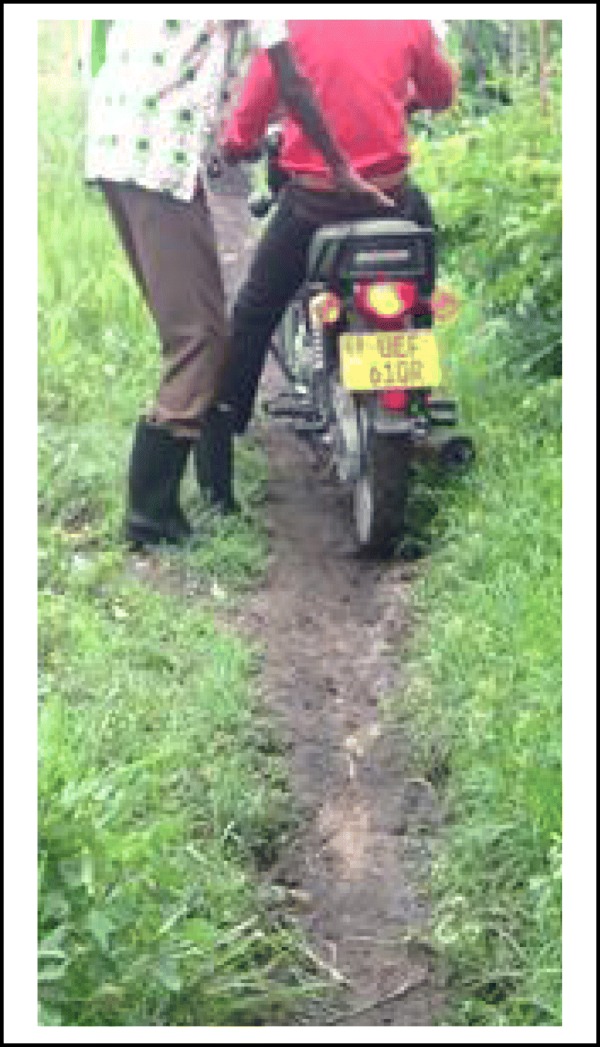
Accessibility to cases

There was a high case fatality rate (CFR) of 1.4% recorded in this outbreak. The high CFR is probably because in Uganda, death of a case or two is usually a common trigger to intervention and surveillance activities. The persistently high number of cases reported in the frequent cholera outbreaks in Kasese district with some deaths at least reported during each outbreak may reflect more general problems in access to effective health care [8, 14]. Uganda enjoys an international applause in management of emergency outbreaks like Ebola and Marburg due to availability of a fully functional state-of-the-art Public Health Emergency Operating Center and an operational public health emergency response plan [15], but more needs to be done in the case of Cholera [16]. Bwera hospital and Kagando hospital, like other hospitals in Uganda, have a small health work force [17] that each time needs to heavily rely on other in-country work force in the emergency situations. Access to effective health care also largely depends on the availability to patients often too ill to walk. Cases were located in areas with unpaved roads (Fig. 6) that could not be accessed by motorized transport means. During the mapping of cases, we went sliding and surviving several falls to access homes of cases. The problem of poorly or unpaved roads though seems to be similar across the resource constrained settings across Africa, Asia and Latin America [18].

Even with the poor road infrastructure, the local district team with the benefit of the previous experience of managing cholera outbreaks quickly and effective responded with awareness messages and active identification of cases. At the time of the outbreak, the laboratory lacked culture media and other necessary supplies necessary to detect and confirm the Cholera cases, at that point, anybody who presented with diarrhea was considered a case. A similar study in Kenya also pointed out similar challenges of supply chain and non-preparedness to respond to cholera outbreaks [19].

Similarly, over 100 people were affected by cholera in the same district in the year 2015 [8]. The frequent outbreaks in Kasese district and other parts of Uganda have continued to feed into the high density of cholera cases reported in Sub-Saharan Africa.

## Environment and sanitation

Kasese District is a hilly area with vast low lands that suffer frequent flooding. The outbreak of 2017 proceeded heavy flooding that washed toilets into the river in the low land. Consumption of contaminated water has been repeatedly implicated in a similar cholera outbreaks in Kasese [8], other parts of the country like Karamoja in North Eastern Uganda [14] and elsewhere in the world [20–22]. Most of the latrines in the affected area were unimproved and this could have encouraged rampant cases of open defecation. While the National Housing and Population census reports that only 10% of the rural population in Uganda lacks access to pit latrines, it also acknowledges that about 58% of the pit latrines are unimproved [23] in the case of Kasese, the District annual report indicates both safe water and latrine coverage are 60%. The quality of latrine facilities needs to be addressed if Kasese is to meet the sanitation SDG targets. Katojo Primary school whose latrine was washed away by the floods also had no hand washing facilities with evidence of open defecation around the latrine which points to the fact that the pupil to stance ratio is higher than the recommended 40:1 (pupil: stance) [24] meaning that the pupils have to queue longer to access the latrine facilities at any given time. Similarly the Uganda national rural school pupil to stance ration has been reported to have increased from 70:1 to 71:1 between 2014 and 2015 [24]. Reducing pupil to stance ratio will help to minimize cases of open defecation in schools which eventually helps to prevent future outbreaks.

### Antibiotic susceptibility

In this study, we report a high resistance to Ampicillin and trimethoprim/Sulfamethoxazole and this compares with the findings of other studies [25]. We note with great concern the high resistance to tetracycline of over 50% yet it is one of the main antibiotics recommended in the management of Cholera (Fig.5). Several other studies have reported the rise in resistance to tetracycline by *V. cholerae* [25, 26]. The greatest susceptibility was noted in gentamicin and low resistance against ciprofloxacin was recorded. Similar studies have documented that there has been no notable change in the resistance to ciprofloxacin [25]. Multi-drug resistance has also been noted in this study. With the emergence of multidrug resistance among *V. cholerae* isolates, Uganda now faces a double challenge of both control and management of cholera epidemics.

## Conclusion

Though we did not culture water to confirm contamination with *Vibrio cholerae*, we hypothesize that the cholera epidemic in Kasese 2017 was sparked off by consumption of contaminated water, following the heavy floods that washed away latines into water sources in Bwera, Isango and Nakiyumbu sub-counties.

V. cholerae was also highly resistant to the commonly used antibiotics.

### Recommendation

Laboratory capacity to detect and monitor the rapidly emerging drug resistance among *V. cholerae* Isolates needs to be improved to effectively handle the dual challenge of treatment and prevention of Cholera. We also recommend proactive surveillance other than reactive surveillance to reduce the case fatality rate and to prevent future outbreaks. Both latrine and safe water coverage need to be improved.

### Generalizability

Our findings can be generalized to similar settings in Uganda and other developing countries.

## Data Availability

All data on which the conclusions of this manuscript are drawn is available on request from the corresponding author.

## Abbreviations

Amp: Ampicillin
CFR: case fatality rate
Chl: Chloramphenicol
Cip: Ciprofloxacin
CLSI: Clinical and Laboratory Standards Institute
Gen: Gentamycin
GPS: Global Positioning System
GWFS: Gravitational Water Flow Schemes SDG Sustainable Development Goals
IDRC: International Development Research Centre Canada
Nal: Nalidixic acid
OHCEA: One Health Central and East Africa
SXT: Trimethoprim/Sulfamethoxazole
Tet: Tetracycline
WHO: World Health Organization
